# Elective one-minute full brain multi-contrast MRI versus brain CT in pediatric patients: a prospective feasibility study

**DOI:** 10.1186/s12880-024-01196-6

**Published:** 2024-01-24

**Authors:** Francesca De Luca, Annika Kits, Daniel Martin Muñoz, Åsa Aspelin, Ola Kvist, Yords Österman, Sandra Diaz Ruiz, Stefan Skare, Anna Falk Delgado

**Affiliations:** 1 Department of Clinical Neuroscience, Karolinska Institute, Stockholm, Sweden; 2 Department of Radiology, Karolinska University Hospital, Stockholm, Sweden; 3 Department of Neuroradiology, Karolinska University Hospital, Stockholm, Sweden; 4 Department of Pediatric Radiology, Karolinska University Hospital, Stockholm, Sweden; 5 Department of Women’s and Children’s Health, Karolinska Institute, Stockholm, Sweden; 6 Department of Radiology, Lund University, Lund, Sweden

**Keywords:** EPIMix, CT, Fast brain MRI, Pediatric brain MRI, Pediatric brain CT

## Abstract

**Background:**

Brain CT can be used to evaluate pediatric patients with suspicion of cerebral pathology when anesthetic and MRI resources are scarce. This study aimed to assess if pediatric patients referred for an elective brain CT could endure a diagnostic fast brain MRI without general anesthesia using a one-minute multi-contrast EPI-based sequence (EPIMix) with comparable diagnostic performance.

**Methods:**

Pediatric patients referred for an elective brain CT between March 2019 and March 2020 were prospectively included and underwent EPIMix without general anesthesia in addition to CT. Three readers (R1–3) independently evaluated EPIMix and CT images on two separate occasions. The two main study outcomes were the tolerance to undergo an EPIMix scan without general anesthesia and its performance to classify a scan as normal or abnormal. Secondary outcomes were assessment of disease category, incidental findings, diagnostic image quality, diagnostic confidence, and image artifacts. Further, a side-by-side evaluation of EPIMix and CT was performed. The signal-to-noise ratio (SNR) was calculated for EPIMix on T1-weighted, T2-weighted, and ADC images. Descriptive statistics, Fisher’s exact test, and Chi-squared test were used to compare the two imaging modalities.

**Results:**

EPIMix was well tolerated by all included patients (*n* = 15) aged 5–16 (mean 11, SD 3) years old. Thirteen cases on EPIMix and twelve cases on CT were classified as normal by all readers (R1–3), while two cases on EPIMix and three cases on CT were classified as abnormal by one reader (R1), (R1–3, *p* = 1.00). There was no evidence of a difference in diagnostic confidence, image quality, or the presence of motion artifacts between EPIMix and CT (R1–3, *p* ≥ 0.10). Side-by-side evaluation (R2 + R4 + R5) reviewed all scans as lacking significant pathological findings on EPIMix and CT images.

**Conclusions:**

Full brain MRI-based EPIMix sequence was well tolerated without general anesthesia with a diagnostic performance comparable to CT in elective pediatric patients.

**Trial registration:**

This study was approved by the Swedish Ethical Review Authority (ethical approval number/ID Ethical approval 2017/2424-31/1). This study was a clinical trial study, with study protocol published at ClinicalTrials.gov with Trial registration number NCT03847051, date of registration 18/02/2019.

**Supplementary Information:**

The online version contains supplementary material available at 10.1186/s12880-024-01196-6.

## Background

Computed tomography (CT) of the brain can be used to evaluate pediatric patients with suspicion of cerebral pathology. However, brain CT has poor soft-tissue contrast [1], exposing the patient to ionizing radiation. Further, radiation exposure from repeated CT examinations in childhood has shown a small but significant effect on the lifetime risk of developing leukemia, brain tumors, and cataract [2–5].

Magnetic resonance imaging (MRI) is a non-ionizing imaging modality with high soft-tissue contrast and detection rate for brain lesions. Although brain MRI is indicated in children with suspected cerebral pathology, some challenges still limit its widespread use in pediatric neuroimaging [6].

Routine clinical MRI traditionally requires long acquisition times, prompting the need for general anesthesia in children presenting with claustrophobia or inability to lay still during the MRI acquisition. Hence, scheduling a brain MRI under sedation or general anesthesia is a time-limiting step in clinical practice. Preferably, pediatric patients should be examined with short MRI acquisitions with sequences robust to motion, limiting the need for general anesthesia.

To overcome the limitations of routine clinical MRI, a multi-contrast echo-planar imaging-based sequence (EPIMix) has recently been developed to provide a full brain MRI scan within a total scan time of 78 s [7]. EPIMix generates six important MRI brain tissue contrasts – T1-weighted, T2-weighted, T2-FLAIR, DWI, ADC, and T2*-weighted. Previous studies in adult patients have demonstrated the feasibility of EPIMix in a variety of clinical settings, ranging from investigating its use in patients with a broad spectrum of cerebral pathologies [8, 9] to diagnosing patients with suspicion of acute cerebral infarction [10, 11].

The study aim was to evaluate the tolerance to undergo an MRI-based EPIMix examination without general anesthesia and its diagnostic performance compared to brain CT in elective pediatric patients.

## Methods

### Participants

Patients referred for an elective brain CT and their caregivers were consecutively asked to participate in this prospective ethical review board-approved study at the Medical Division of Neuroradiology and Pediatric Radiology, Karolinska University Hospital, from March 2019 to March 2020. Inclusion criteria were patients aged 4–18 years old with non-acute symptoms referred for an elective brain CT scheduled after 14 days or more. Non-acute CT referrals were chosen to not interfere with or delay acute investigations but rendered referrals with a low suspicion of cerebral pathology. Exclusion criteria were an indication of acute brain CT, canceled or rescheduled examinations, or inaccurate image reconstruction. Relevant medical history and neurological examination data were retrieved from referrals and medical charts. This study was approved by the Swedish Ethical Review Authority (ethical approval number/ID Ethical approval 2017/2424-31/1). Informed consent from all the subjects and subjects’ parents or legal guardians was obtained included in the study. Images and information in this study were anonymized and presented as not identifiable. This study was approved by the Swedish Ethical Review Authority (ethical approval number/ID Ethical approval 2017/2424-31/1). This study was a clinical trial study, with study protocol published at ClinicalTrials.gov with Trial registration number NCT03847051, date of registration 18/02/2019.

### Image acquisition

#### CT imaging

Included patients underwent a non-contrast CT scan as part of the clinical routine on a Siemens Somatom Force scanner (Siemens Healthineers, Forchheim, Germany). The technical parameters for the CT protocol were axial acquisition with 120 kV and mAs of approximately 190 mAs covering the skull base to the vertex. The FOV was set to 200 mm with a 512 × 512 matrix. The isovolumetric voxels (1 mm) raw data were post-processed with bone and soft tissue kernels in axial, coronal, and sagittal planes with 3 mm slice thickness.

#### MR Imaging

Brain MRI was performed with EPIMix in a SIGNA 3 T, GE (GE Healthcare, Milwaukee, WI) MRI system. Six axial echo-planar imaging-based MR contrasts were obtained (T1-FLAIR, T2WI, T2-FLAIR, DWI, ADC, T2*WI) using 4 mm slice thickness, 240 mm FOV, 180 × 180 matrix, an acceleration factor of *R* = 3 and a total acquisition time of 78 s. Detailed information about the EPIMix sequence has been previously published [7].

#### Image analysis

EPIMix and CT scans were anonymized and independently evaluated on two separate occasions by a pediatric neuroradiologist (reader 1: R1) with 15 years of pediatric neuroradiology experience, a radiology resident (reader 2: R2) with two years of radiology experience, and a neuroradiologist (reader 3: R3) with seven years of neuroradiology experience. To avoid recall bias favoring EPIMix, R1 and R2 analyzed the CT examinations first. To evaluate if results varied depending on the reading order, R3 analyzed EPIMix examinations first. Additionally, the first reading was followed by a memory washout interval of at least two weeks. The readers were blinded to referral information, health records, and radiology reports. The primary and secondary outcomes were prospectively predefined and included in the prospectively published image analysis protocol before the inclusion of patients.

The primary study outcome was the tolerance to undergo an EPIMix investigation without general anesthesia. The co-primary outcome was to classify the examination as normal or abnormal. Secondary study outcomes included disease category, incidental findings, diagnostic image quality, diagnostic confidence, image artifacts, and clinical recall bias in the evaluation. In addition to the blinded analysis, unblinded side-by-side evaluations of EPIMix and CT scans were performed by R2 together with two additional experienced neuroradiologists (reader 4: R4, reader 5: R5) for the outcomes “scan classification”, “disease category”, and “incidental findings”. The signal-to-noise ratio (SNR) was calculated for EPIMix on T1-weighted, T2-weighted, and ADC images. Two pairs of regions of interest (ROI) were drawn in each hemisphere, in the frontal cortex, frontal white matter, thalamus, and in the cerebral spinal fluid (CSF) at the level of the posterior horn of the lateral ventricles. Two ROIs were also drawn outside the skull, serving as background. T1 background ROI was used for T1-weighted images, whereas T2 background ROI for T2-weighted and ADC images. The signal (S) was calculated as the average ((right + left)/2) of the mean pixel intensity in the different tissues. The noise (N) was calculated as the average ((right + left)/2) of the standard deviation of the signal intensity in the background ROIs. The SNR formula was SNR = 0.655 x (S/N) [12].

### Statistical analysis

The completion rate for EPIMix was calculated as the ratio between completed and attempted exams.

After image analysis, results were dichotomized:

- Scan classification:
  - Likert Scale points 1–2 = normal
  - Likert Scale points 3–5 = abnormal
- Diagnostic image quality:
  - Likert Scale points 1–3 = good
  - Likert Scale point 4 = bad
- Diagnostic confidence:
  - Likert Scale points 1–2 = confident
  - Likert Scale points 3–4 = not confident
- Image artifacts:
  - “not present” plus “present but not degrading” = no degrading artifacts
  - “present and degrading” = degrading artifacts

Differences between EPIMix and CT in scan classification, diagnostic image quality, diagnostic confidence, and image artifacts between readers were evaluated using descriptive statistics, Fisher’s exact test, and Chi-squared test. Contingency tables were used to summarize disease categories and incidental findings. Statistical analysis was performed using GraphPad Prism (GraphPad Software v 9.3, San Diego, California USA) and MATLAB (The Mathworks Inc., R2021a).

## Results

**Fig. 1:**
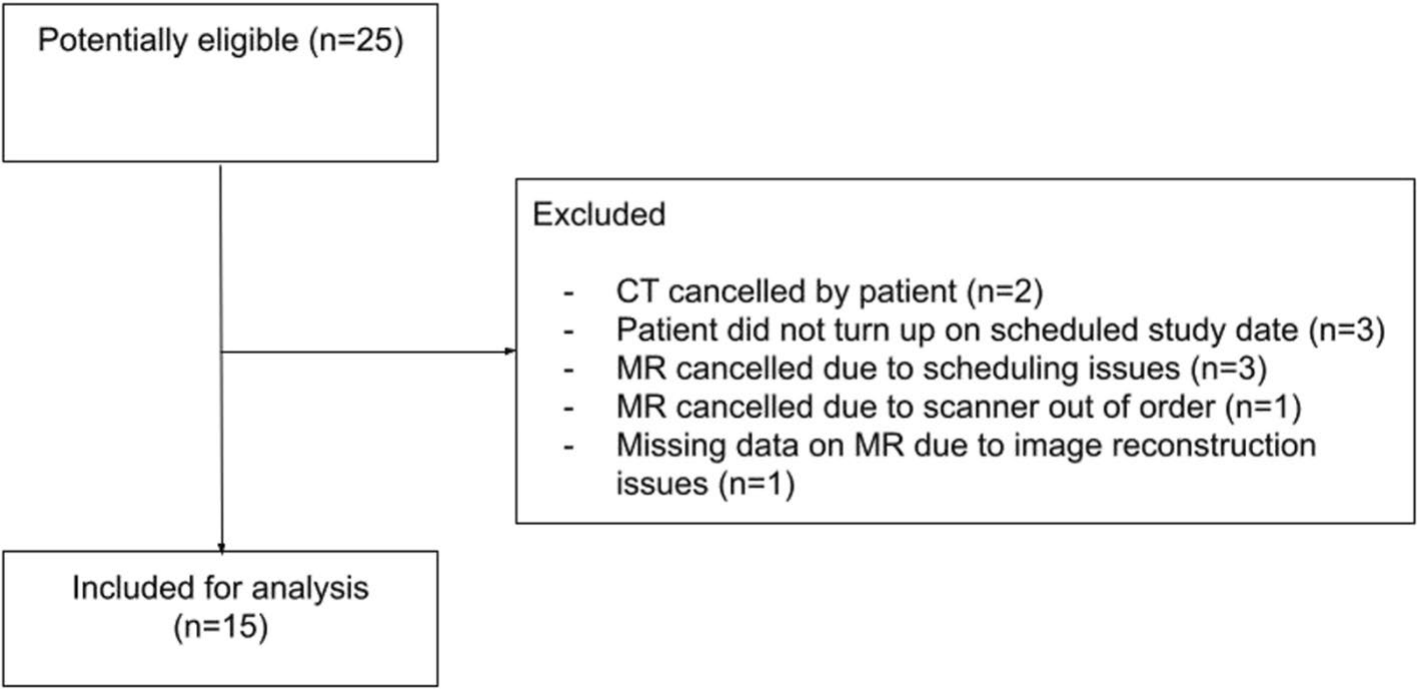
Enrollment chart

Of the potentially eligible 30 patients referred for an elective CT during the study inclusion period, 25 patients consented to participate in the study (25/30, 83%). After the exclusion of nine patients due to scheduling issues and one patient with MR image reconstruction problems, a total of 15 patients were included in the study. Detailed information about patient enrollment can be found in Fig. 1, with patient characteristics summarized in Table 1.

**Table 1:** Patients’ characteristics

Study participants, n 15	
Age years mean (SD, range)	11 (3, 5–16)
Sex female, n (%)	13 (87)
Reported symptoms before examination^a^, n (%)	
Headache	15 (100%)
Nausea or vomiting	3 (20%)
Dizziness	1 (7%)
Syncope	1 (7%)
Clinical queries in the referral, n (%)	
Brain tumor	13 (87%)
Vascular pathology	1 (7%)
Increased intracranial pressure	2 (13%)
Hydrocephalus	1 (7%)
Residual brain injury after previous bleeding	1 (7%)
Unspecified suspicion of cerebral pathology	5 (33%)
Scan time delay between CT and EPIMix acquisition post-contrast, median time in minutes	34
EPIMix acquired prior CT, n (%)	3 (20%)
CT acquired prior EPIMix, n (%)	12 (80%)

*SD* standard deviation

^a^The neurological examination was normal for all patients

### Primary outcome - tolerance to undergo EPIMix without general anesthesia

The EPIMix acquisition was successfully completed without general anesthesia and generated images in 15 out of 15 patients (15/15).

### Co-primary outcome - scan classification

**Fig. 2:**
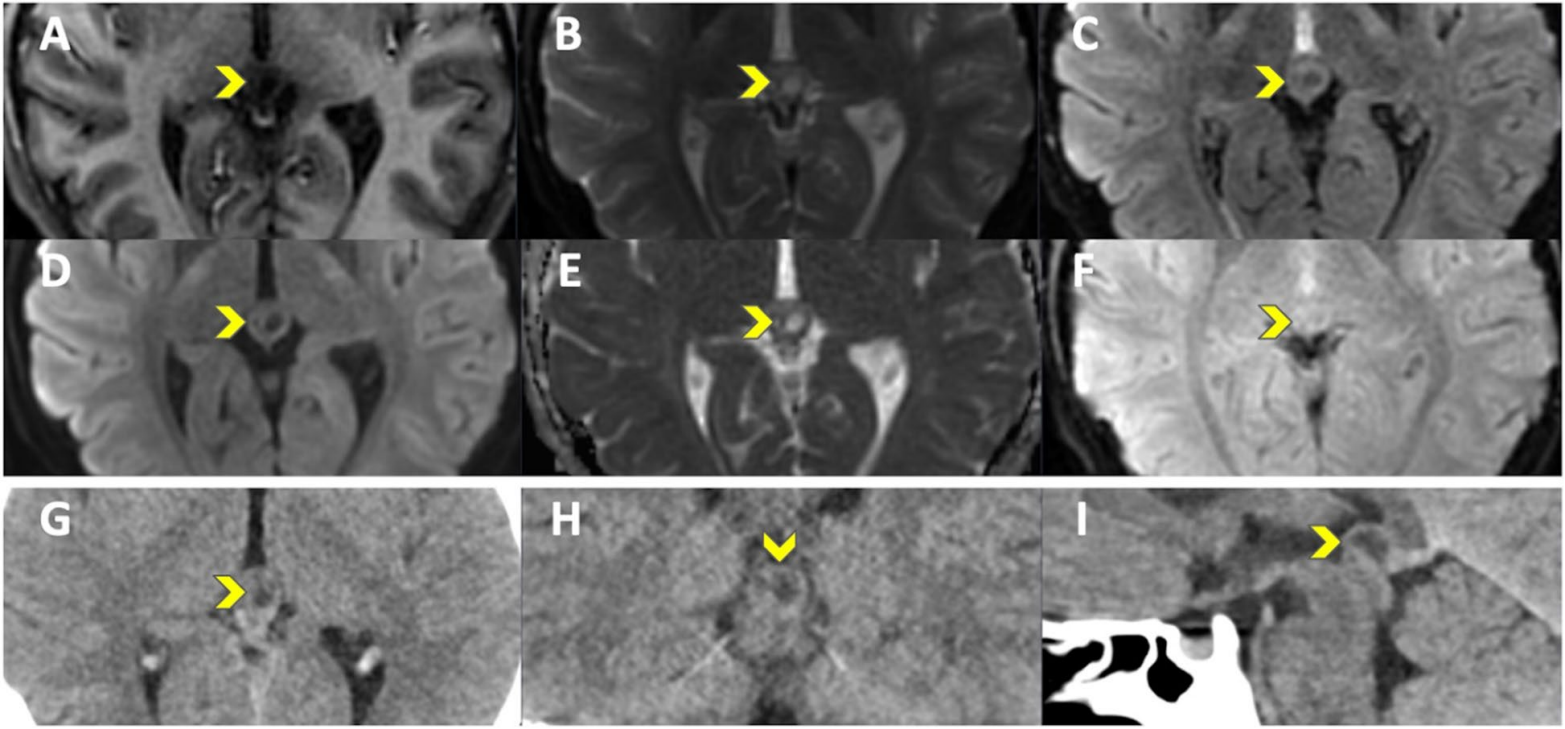
ID2. A pineal cyst (yellow head arrow), visible on both EPIMix and CT at the side-by-side consensus. T1-FLAIR (**A**), T2 (**B**), T2-FLAIR (**C**), DWI (**D**), ADC (**E**), T2* (**F**) EPIMIX images, and axial (**G**), coronal (**H**), sagittal (**I**) soft tissue window CT images

R1 classified two patients (ID1 and 2) on EPIMix and three patients on CT (ID2 Fig. 2, ID11 and ID15) as abnormal, while the other readers (R2 and R3) reported no abnormal scans. There was no evidence of a difference between EPIMix and CT within each reader, Fisher’s exact test *p* = 1.00, R1–3. Side-by-side evaluation (R2, R4–5) classified all scans as lacking significant pathological findings on EPIMix and CT images. Detailed results of scan classification can be found in Table 2. Results before dichotomization can be found in Supplementary Fig. 1.

**Table 2:** Results after dichotomization for the outcomes “scan classification” and “incidental findings”

	Study reading	Study reading	Side-by-side	Side-by-side
**Pat ID**	**EPIMix (R1, R2, R3) significant finding / incidental finding**	**CT (R1, R2, R3) significant finding / incidental finding**	**EPIMix (R2, R4, R5) significant finding / incidental finding**	**CT (R2, R4, R5) significant finding / incidental finding**
1	1, 0, 0 / 1^a^, 0, 1^b^	0, 0, 0 / 1^c^^d^, 0, 0	0 / 1^a^^b^^c^	0 / 1^d^
2	1^e^, 0, 0 / 0, 1^e^, 1^e^	1^e^, 0, 0 / 1^f^, 1^f^^g^, 0	0 / 1^a^^e^^f^^g^	0 / 1^e^^f^^g^
3	0, 0, 0 / 1^a^, 0, 0	0, 0, 0 / 0, 0, 0	0 / 1^a^	0 / 0
4	0, 0, 0 / 1^h^, 0, 0	0, 0, 0 / 0, 1^g^^i^, 0	0 / 1^a^	0 / 1^g^
5	0, 0, 0 / 1^a^^h^, 0, 1^c^	0, 0, 0 / 1^j^, 0, 0	0 / 1^a^^c^	0 / 0
6	0, 0, 0 / 1^a^, 0, 1^c^	0, 0, 0 / 1^a^^k^, 1^g^, 0	0 / 1^a^^c^^k^	0 / 1^g^^k^
7	0, 0, 0 / 0, 0, 0	0, 0, 0 / 1^a^^j^, 0, 0	0 / 1^a^	0 / 1^a^
8	0, 0, 0 / 0, 0, 0	0, 0, 0 / 0, 1^g^, 0	0 / 1^a^^g^	0 / 1^g^^l^
9	0, 0, 0 / 1^a^, 0, 0	0, 0, 0 / 0, 0, 0	0 / 1^a^	0 / 0
10	0, 0, 0 / 1^a^, 0, 0	0, 0, 0 / 1^c^, 0, 0	0 / 1^a^^c^	0 / 1^a^
11	0, 0, 0 / 0, 0, 0	1^j^, 0, 0 / 1^c^, 0, 1^j^	0 / 1^a^	0 / 0
12	0, 0, 0 / 1^a^, 0, 1^b^	0, 0, 0 / 1^a^, 1^l^, 0	0 / 1^a^^b^	0 / 1^a^^l^
13	0, 0, 0 / 0, 0, 0	0, 0, 0 / 0, 0, 0	0 / 0	0 / 0
14	0, 0, 0 / 0, 0, 0	0, 0, 0 / 0, 1^g^, 1^g^^l^	0 / 1^g^	0 / 1^g^^l^
15	0, 0, 0 / 1^a^^m^, 1^e^, 0	1^e^, 0, 0 / 1^n^, 0, 0	0 / 1^a^^e^	0 / 1^e^

Scan classification (significant finding) / Incidental finding: 0 = no; 1 = yes

Findings:

^a^Midline CSF-filled cavities (cavum vergae, cavum septi pellucidi, cavum veli interpositi)

^b^Developmental venous anomaly

^c^Virchow-Robin spaces

^d^Dilated vein

^e^Pineal cyst

^f^Thickened gyrus (pachygyria) with overlying calvarial cortical thinning

^g^Paranasal sinus mucosal thickening

^h^Bilateral high signal in occipital white matter

^i^Tentorial calcification

^j^Wide sulcus

^k^Focal ventricular dilatation

^l^ Fluid level in paranasal sinuses

^m^High signal in periaqueductal gray matter

^n^Tonsillar ectopia

### Secondary outcomes

#### Disease category

**Fig. 3:**
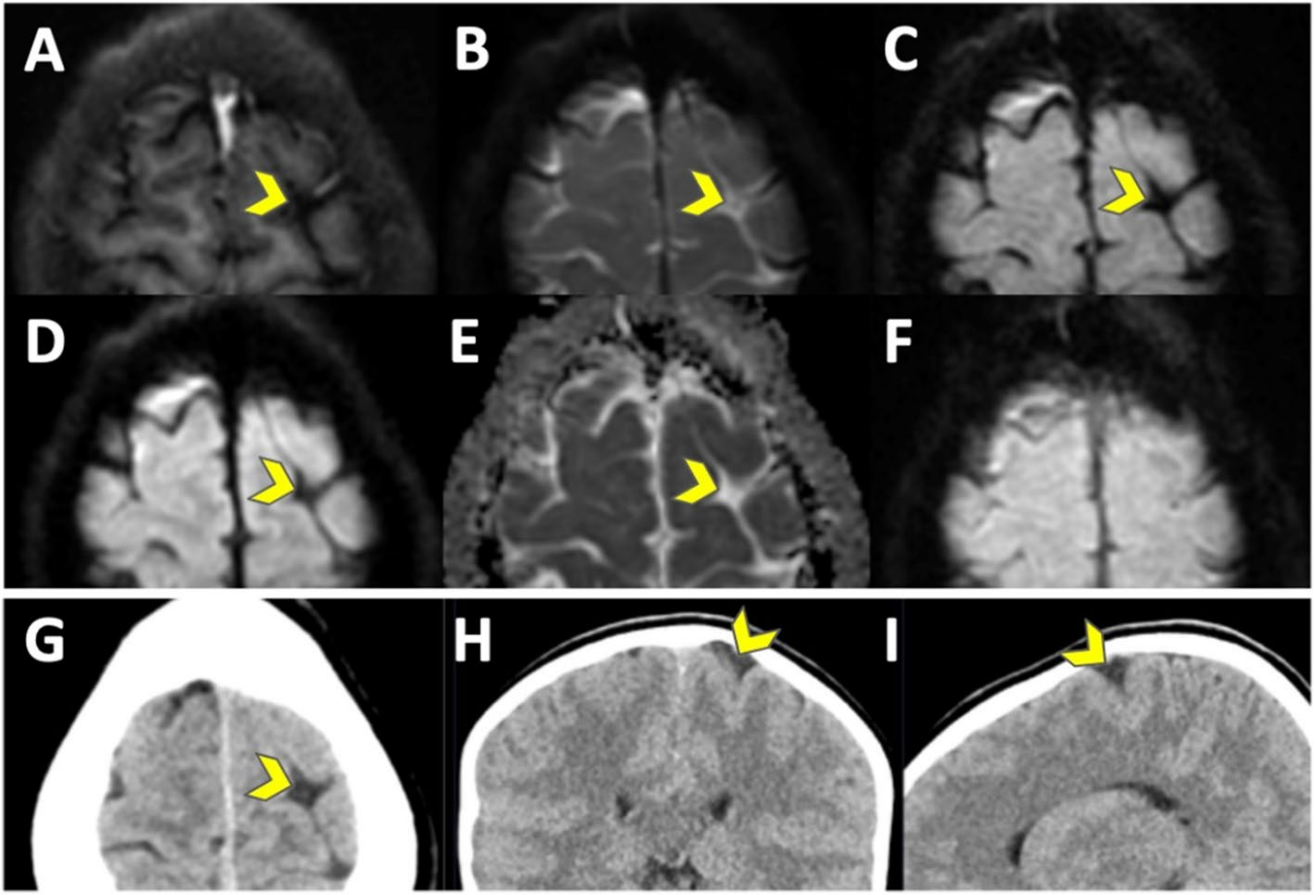
ID11. A wide sulcus (yellow arrowhead) reported together with suspected underlying focal cortical dysplasia, the latter not confirmed at the side-by-side consensus. T1-FLAIR (**A**), T2 (**B**), T2-FLAIR (**C**), DWI (**D**), ADC (**E**), T2* (**F**) EPIMIX images, and axial (**G**), coronal (**H**), sagittal (**I**) soft tissue window CT images

R1 reported one scan as neoplastic (ID2, pineal gland cyst, Fig. 2) and one as indeterminate (ID1) on EPIMix. On CT, R1 reported two scans as neoplastic (ID2 and ID15, pineal gland cyst) and one as malformation (ID11 Fig. 3, wide sulcus) on CT. R2 and R3 reported all MRI and CT scans as normal, although with incidental findings, summarized in Table 2. Unblinded side-by-side evaluation (R2, R4, R5) reviewed all EPIMix and CT scans in consensus as normal with incidental findings, Supplementary Table 1.

#### Incidental findings - free-text report

**Fig. 4:**
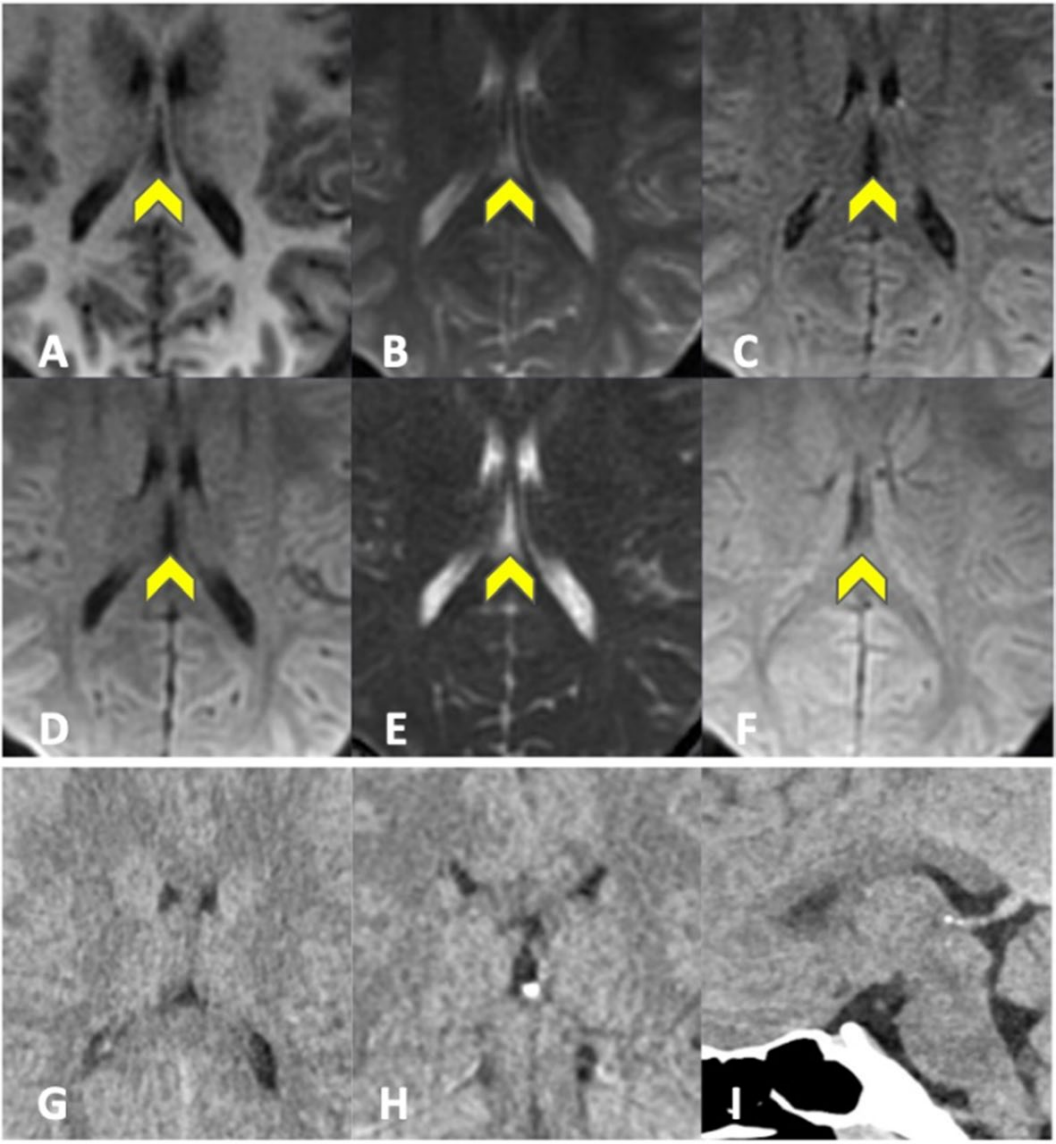
ID3. Midline CSF-filled cavities (yellow head arrow), visible only on EPIMix (side-by-side consensus). T1-FLAIR (**A**), T2 (**B**), T2-FLAIR (**C**), DWI (**D**), ADC (**E**), T2* (**F**) EPIMIX images, and axial (**G**), coronal (**H**), sagittal (**I**) soft tissue window CT images for reference

**Fig. 5:**
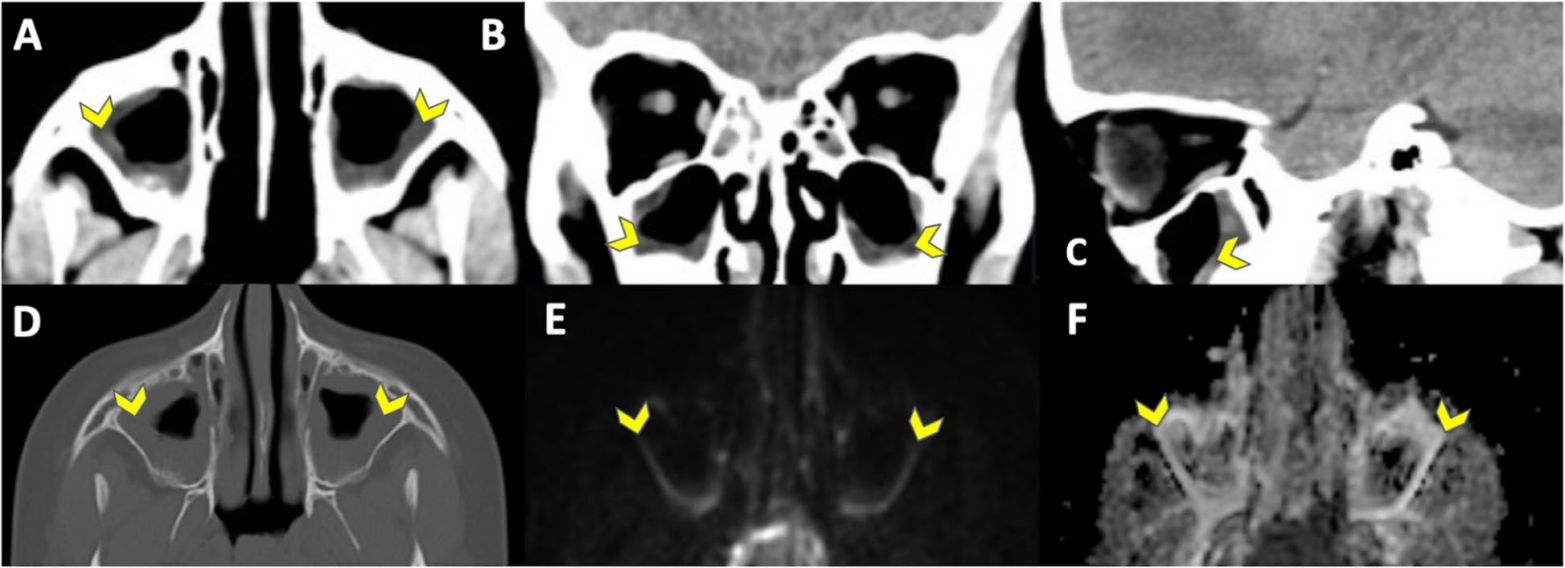
ID14. Paranasal sinus mucosal thickening (yellow head arrow), visible on both EPIMix and CT at the side-by-side consensus. Axial (**A**), coronal (**B**), sagittal (**C**) soft tissue window CT images, axial (**D**) bone window CT image, and DWI (**E**), ADC (**F**) EPIMIX images

Blinded readers (R1–3) reported incidental findings in a total of 10 patients on EPIMix and 12 patients on CT (Table 2). The findings ranged from pineal cysts (EPIMix) to paranasal sinus mucosal thickening (CT). Side-by-side evaluation of incidental findings reported non-significant pathology in 14 out of 15 patients on EPIMix (93%) and in 10 out of 15 patients on CT (67%) (Supplementary Table 1), Supplementary Figs. 2 and 3. In accordance with the blinded evaluation, most of the incidental findings on EPIMix were midline CSF-filled cavities (13/15, 87%, Fig. 4, Supplementary Fig. 4). In accordance with CT, and as opposed to the blinded evaluation of EPIMix, paranasal sinus mucosal thickening was also evident on EPIMix at side-by-side evaluation, Fig. 5. However, the fluid levels reported in two patients could not be discerned on EPIMix despite being evident on CT at the side-by-side consensus evaluation.

#### Diagnostic image quality, diagnostic confidence

**Fig. 6:**
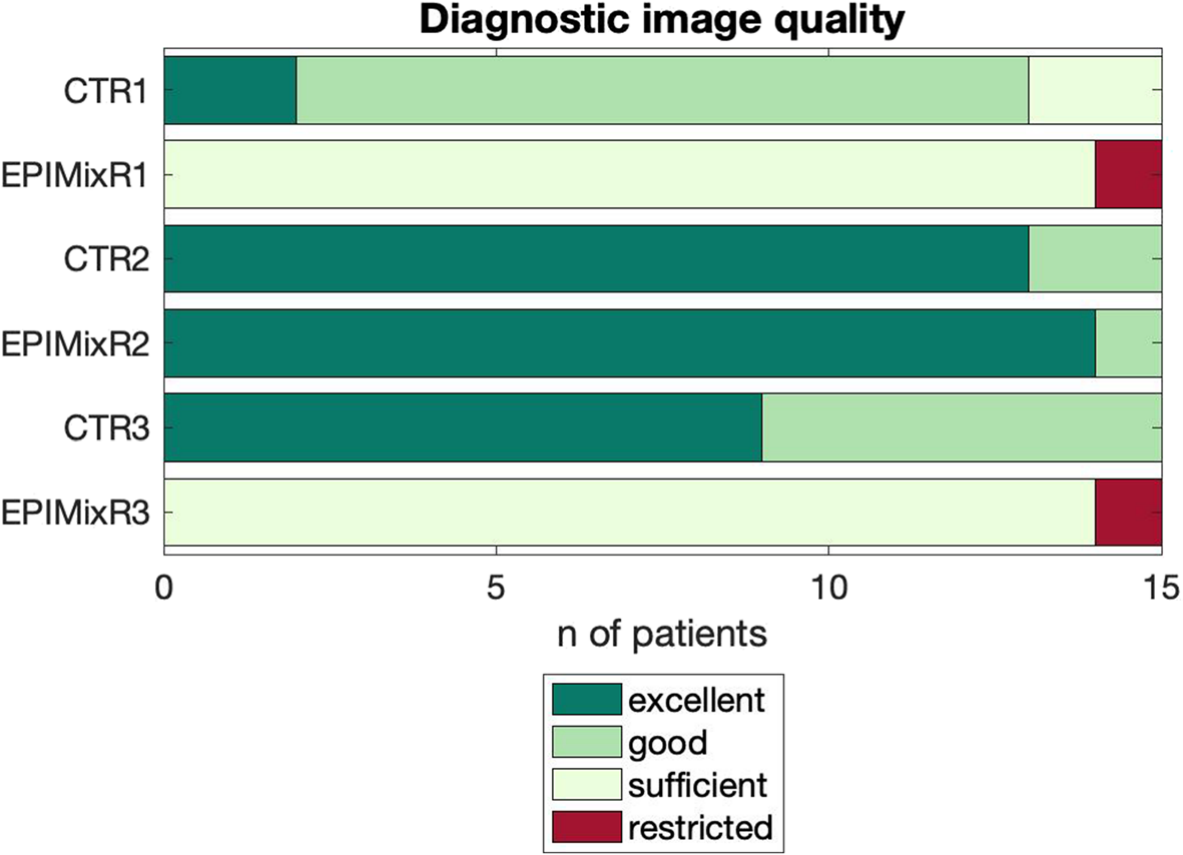
Diagnostic image quality on EPIMix and CT scans on a per-reader (1 –3)  basis (CTR – CT reader, EPIMixR – EPIMixreader)

**Fig. 7:**
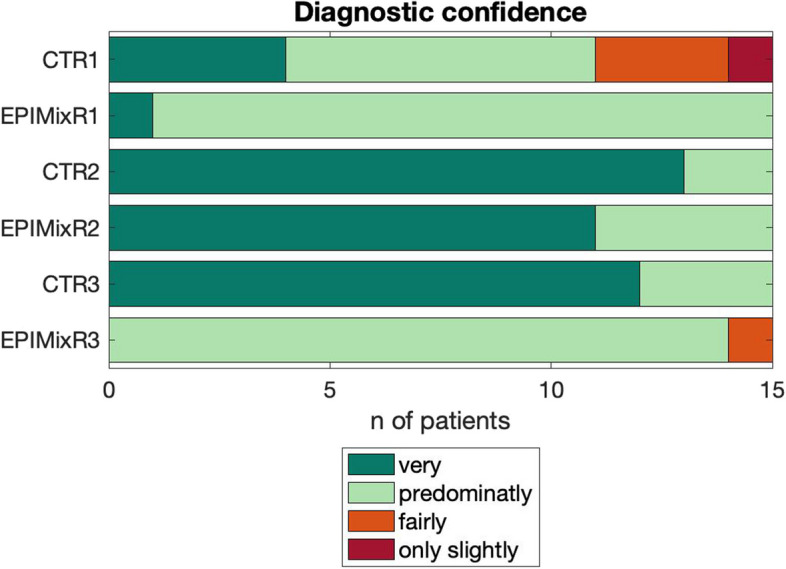
Diagnostic confidence on EPIMix and CT scans on a per-reader (1–3) basis (CTR – CT reader, EPIMixR – EPIMixreader)

After dichotomization, there was no evidence of a difference in diagnostic image quality (good/bad) and diagnostic confidence (confident/not confident) between EPIMix and CT on a per-reader basis (R1–3, Fisher exact test *p* ≥ 0.10). Results before dichotomization can be found in Figs. 6 and 7. Two cases of EPIMIX were evaluated as presenting with “restricted” image quality, Supplementary Figs. 5 and 6. The diagnostic confidence for EPIMIX was “predominantly confident” in a majority of the cases read by R1 and R3 (14/15) and “very confident” for R2 (11/15). The diagnostic confidence for CT was graded as “fairly confident” in four cases (R1, 3/15; R3, 1/15) and as “only slightly confident” in one case (R1, 1/15, 7%).

#### Image artifacts

After dichotomization, there was no evidence of a difference in motion artifacts between EPIMix and CT on a per-reader basis R1–3, Fisher exact test *p* = 1.00. Motion artifacts were reported in a majority of cases as “not present” or “present but not degrading” by R1–3 (EPIMix R1–3 14/15 93%; CT: R1,3 15/15 100%, R2 13/15 87%). One case of “degrading” motion artifacts was reported on EPIMix by all readers (R1–3, 1/15, 7%), and one case with “degrading” motion artifacts was reported by one reader on CT (R2, 1/15, 7%), Supplementary Fig. 7.

Beam hardening artifacts were reported in a majority of cases as “not present” or “present but not degrading” (R1 14/15 93%, R2–3 15/15 100%), with one case reported with “degrading” beam hardening artifacts at the skull base (R1, 1/15, 7%), Chi-squared test interrater *p* = 0.36 R1–3, Supplementary Fig. 8.

After dichotomization, a significant difference was found in the assessment of susceptibility distortion on EPIMix interrater R1–R3. Susceptibility distortion artifacts were evaluated as “present and degrading” in 93% (R1 14/15), 0% (R2 0/15), and 100% (R3 15/15), Chi-squared test interrater *p* < 0.0001, Supplementary Fig. 9. In detail, the reported “degrading” susceptibility distortion artifacts on EPIMix were described as artifacts at the posterior fossa/skull base (R1, 14/15, 93%; R3 15/15, 100%), and right/left distortion (R1 1/15, 7%). Further, cerebrospinal fluid flow artifacts were also separately reported by one reader (R1 8/15, 53%).

#### Recall bias evaluation

No recall bias in the assessments was reported (R1–3).

### EPIMix SNR values

The SNR values for EPIMix on T1-weighted, T2-weighted, and ADC images are summarized in Supplementary Table 2.

## Discussion

This prospective study aimed to compare a new fast MRI technique EPIMix to brain CT for pediatric patients referred for an elective investigation with a relatively low suspicion of cerebral pathology. The new method EPIMix was well tolerated without general anesthesia. Further, EPIMix showed a comparable diagnostic performance to CT.

The study results indicate that, in this specific patient group, a brain CT can be replaced by a short MRI scan without impeding the diagnostic performance. Replacing CT with EPIMix would lower the patient’s radiation exposure from CT and is especially important in children undergoing repeated examinations. The importance of not exposing children to ionizing radiation from CT is higher if the pre-test probability of disease is low, as in this study [13]. In this study, including non-acute examinations scheduled after more than 14 days, significant imaging findings were scarce, and the clinical suspicion to detect cerebral pathology was low [14]. When an alternative method without ionizing radiation such as EPIMix exists, it is harder to justify a CT investigation. Despite the scheduling of clinical CT examinations after two weeks from referral, the clinical question in a majority of patients, as stated in the referral, was “brain tumor?”. Previous studies have investigated the clinical feasibility of fast MRI methods in pediatric patients with hydrocephalus [15, 16], acute arterial ischemic stroke [17], traumatic brain injury [18], and non-traumatic pediatric emergency [19], while others have evaluated the diagnostic image quality of fast brain MRI [20–22] and fast spine MRI [23]. In accordance with the results from these previous studies, EPIMix performed well in a clinical situation, showing comparable diagnostic performance to CT in pediatric patients. Included patients in this study had a non-acute headache as the primary neurological manifestation, with a minority of cases presenting nausea/vomiting, dizziness, and syncope. Similarly, previous radiological studies on pediatric patients reported headache [15, 17, 19–21, 23], vomiting [15, 19–21], and syncope [19, 21], but also altered mental status [15, 17, 19], seizures [15, 17, 19–21], and pain [15].

Previous studies on EPIMix in adult patients have investigated a variety of neurological conditions, such as ischemic stroke [10, 11], patients with suspicion of brain pathology [8], and the clinical feasibility of the sequence [9, 24]. These studies showed comparable diagnostic performance [8, 10, 11] and sufficient image quality [9] for EPIMix compared to routine brain MRI. Not surprisingly, EPIMix also performed well against CT in the current study.

An advantage compared to some of the previous retrospective studies [15–17, 19, 21, 23] is the prospective design of the present work. Further advantages of EPIMix compared to previous studies is the acquisition of a higher number of tissue contrasts, as opposed to only T2-weighted [15, 22], T2 + T2* [18], T2 + T1 [23], and T2 + DWI [17]. Further, EPIMix has a shorter acquisition time than most previous studies with scan times ranging from 2 up to 22 min [15, 17–20, 22, 23]. Also, comparing EPIMix to CT is important since it might obviate the need for a CT if a short MRI scan can answer the clinical question with similar diagnostic performance. Assuming a roughly equal scanning time and no sedation for CT or EPIMix, the healthcare cost would be similar.

EPIMix presented more artifacts than CT, with susceptibility distortion artifacts being the most common. This is unsurprising as the method is EPI-based with well-known technical issues but an unsurpassed MRI acquisition speed. Despite these artifacts, the diagnostic confidence for EPIMix was not largely affected, with diagnostic performance comparable to CT.

Image evaluation was performed by three blinded readers with different levels of experience, as recommended for multi-reader studies [25]. Despite the divergent experience among readers, there was no evidence of a difference in scan classification, diagnostic image quality, confidence, and motion artifacts evaluation between EPIMix and CT, which strengthens the generalizability of the results. SNR ranges for T1-weighted and T2-weighted images in this study are in line with those previously reported in the literature [12]. Previous studies in the field of fast MRI had fewer readers [15, 18, 20, 21], readers with similar radiology experience [15, 22], non-radiology and expert pediatric neuroradiology readers [19], or information about fast MRI obtained through a questionnaire or clinical charts including radiology reports [16, 17, 23].

One limitation of the study was the small sample size, primarily due to the exploratory nature of this study and logistical resource-based challenges that hampered the recruitment of a higher number of subjects. Another limitation was that all included subjects referred for an elective CT had normal neurological status and, hence a lower grade of suspicion for cerebral pathology compared to patients with neurological symptoms. As a direct consequence, all included patients presented with normal or incidental findings at neuroimaging. Further studies are needed to evaluate EPIMix in pediatric patients with significant cerebral pathology and determine whether EPIMix can replace CT to rule out or confirm such pathology. Additionally, although not specifically investigated in this study, EPIMix has previously shown a higher number of image artifacts compared to conventional MRI [8]. Finally, the study population had a skewed sex ratio, reflecting the known higher prevalence of headache observed in females [26].

## Conclusions

Full brain MRI-based method EPIMix was well tolerated without general anesthesia with a diagnostic performance comparable to CT. EPIMix might be a feasible imaging alternative to elective brain CT in pediatric patients.

### Supplementary information

**Additional file 1.**

## Data Availability

Aggregated and anonymized data used or analyzed during the current study are available from the corresponding author, Francesca De Luca, upon reasonable request.

## Abbreviations

EPIMix: multi-contrast echo planar imaging-based sequence
R1: Reader 1
R2: Reader 2
R3: Reader 3
R4: Reader 4
R5: Reader 5
