# Supplementary material for: Elective one-minute full brain multi-contrast MRI versus brain CT in pediatric patients: a prospective feasibility study
Source: BMC Med Imaging. 2024 Jan 24;24:23. doi: 10.1186/s12880-024-01196-6 (PMC10809606; doi:10.1186/s12880-024-01196-6)

Elective One-Minute Full Brain Multi-Contrast MRI versus Brain CT in Pediatric Patients: A Prospective Feasibility Study

Supplementary Table 1. Pivot table for reported findings by blinded readers and side-by-side evaluation.

| **EPIMix** |  |  | Side-by-side | Side-by-side | **CT** |  |  | Side-by-side | Side-by-side |
| --- | --- | --- | --- | --- | --- | --- | --- | --- | --- |
|  |  |  | Significant finding | Significant finding |  |  |  | Significant finding | Significant finding |
|  |  |  | Yes | No |  |  |  | Yes | No |
| Blinded readers | Significant finding (any reader) | Yes | 0 | 2 | Blinded readers | Significant finding (any reader) | Yes | 0 | 3 |
| Blinded readers | Significant finding (any reader) | No | 0 | 13 | Blinded readers | Significant finding (any reader) | No | 0 | 12 |
| **EPIMix** |  |  | Side-by-side | Side-by-side | **CT** |  |  | Side-by-side | Side-by-side |
|  |  |  | Incidental finding | Incidental finding |  |  |  | Incidental finding | Incidental finding |
|  |  |  | Yes | No |  |  |  | Yes | No |
| Blinded readers | Incidental finding (any reader) | Yes | 10 | 0 | Blinded readers | Incidental finding (any reader) | Yes | 10 | 2 |
| Blinded readers | Incidental finding (any reader) | No | 4 | 1 | Blinded readers | Incidental finding (any reader) | No | 0 | 3 |

Supplementary Table 2. SNR mean values (range) for EPIMix on T1-weighted, T2-weighted, and ADC images.

| mean (range) | SNR cortex | SNR white matter | SNR thalamus | SNR CSF |
| --- | --- | --- | --- | --- |
| T1-weighted | 84.2 (36.4–156) | 182.4 (76.4–284.1) | 121 (44.7–206.5) | 28.6 (9–74.8) |
| T2-weighted | 155 (79.4–232.7) | 99.7 (55.8–159.4) | 112.7 (69.2–176.3) | 292 (164.9–445.2) |
| ADC | 136.1 (43.1–254.8) | 120.9 (42.8–256.9) | 112.8 (38.7–203.8) | 407.9 (148.2–735.9) |

Supplementary Figure 1. Scan classification on EPIMix and CT scans on a per-reader (1–3) basis (CTR – CT reader, EPIMixR – EPIMixreader).


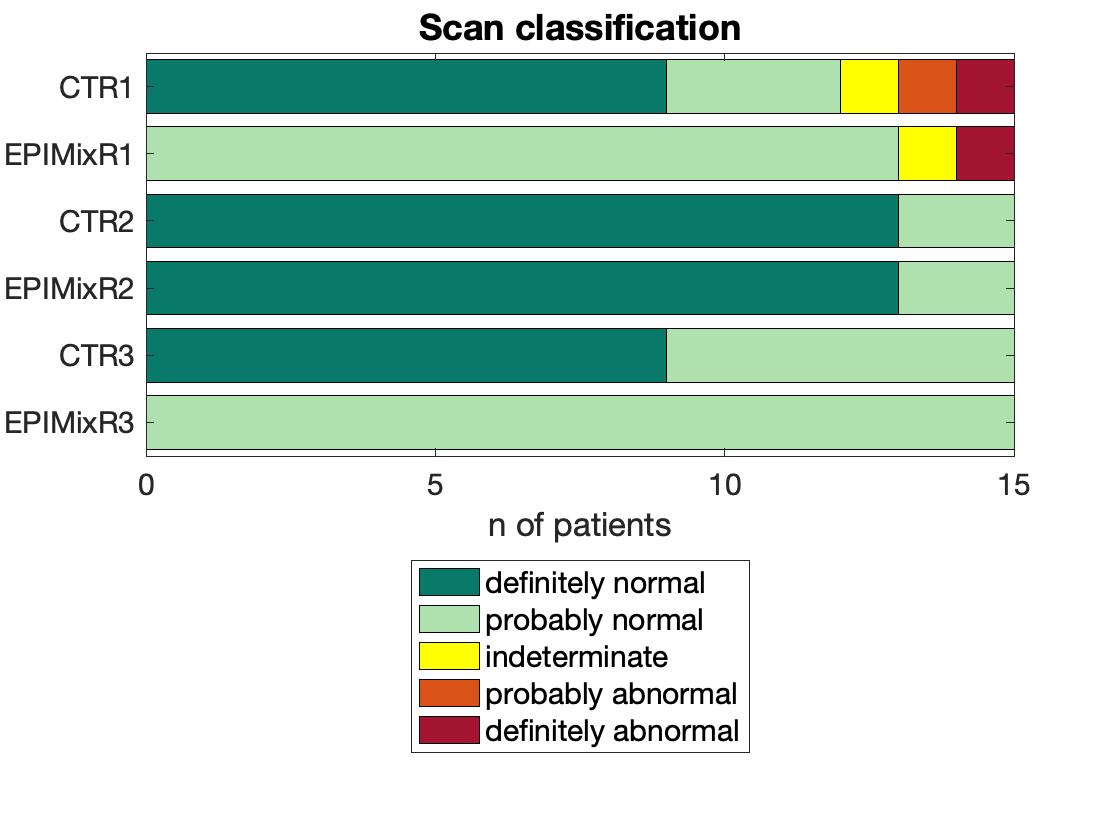


Supplementary Figure 2. ID4. Bilateral high signal in the occipital white matter (yellow head arrow), deemed as an artefact on EPIMix at the side-by-side consensus. T1-FLAIR (A), T2 (B), T2-FLAIR (C), DWI (D), ADC (E), T2* (F) EPIMIX images.


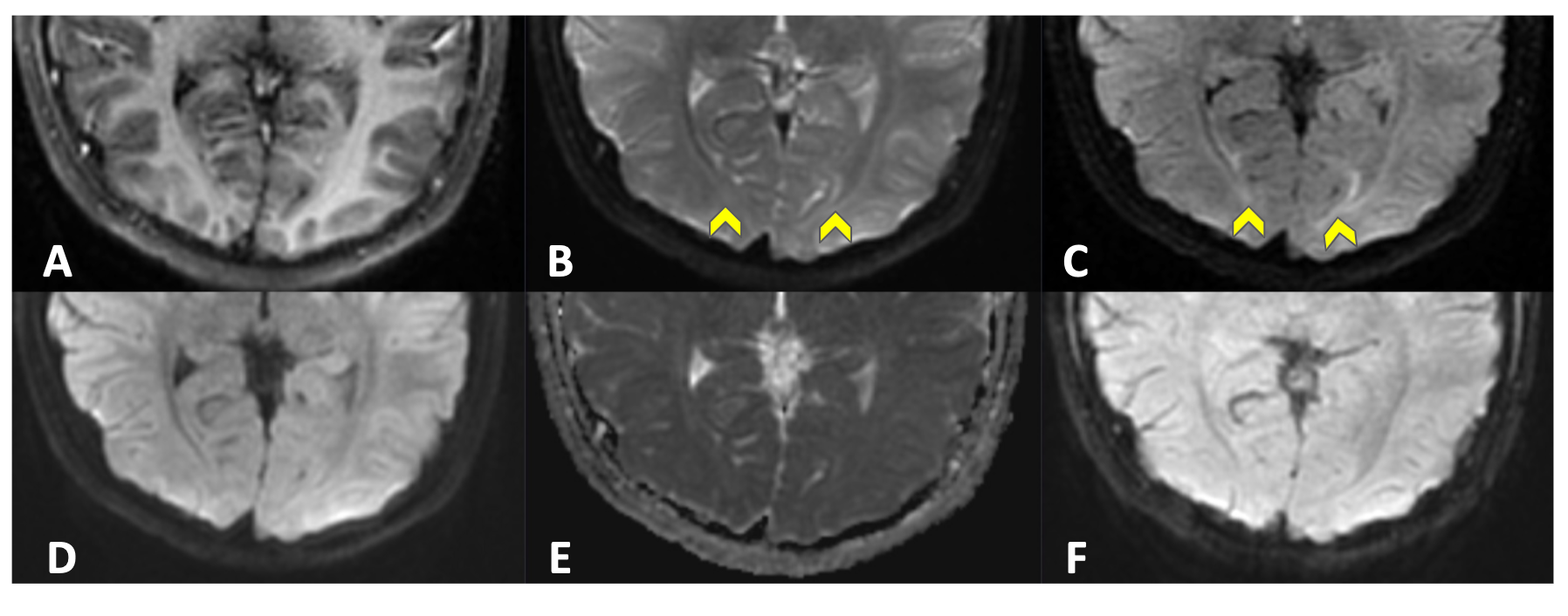


Supplementary Figure 3. ID6. Virchow-Robin space (yellow head arrow), visible only on EPIMix at the side-by-side consensus. T1-FLAIR (A), T2 (B), T2-FLAIR (C), DWI (D), ADC (E), T2* (F) EPIMIX images, and axial (G), coronal (H), sagittal (I) soft tissue window CT images.


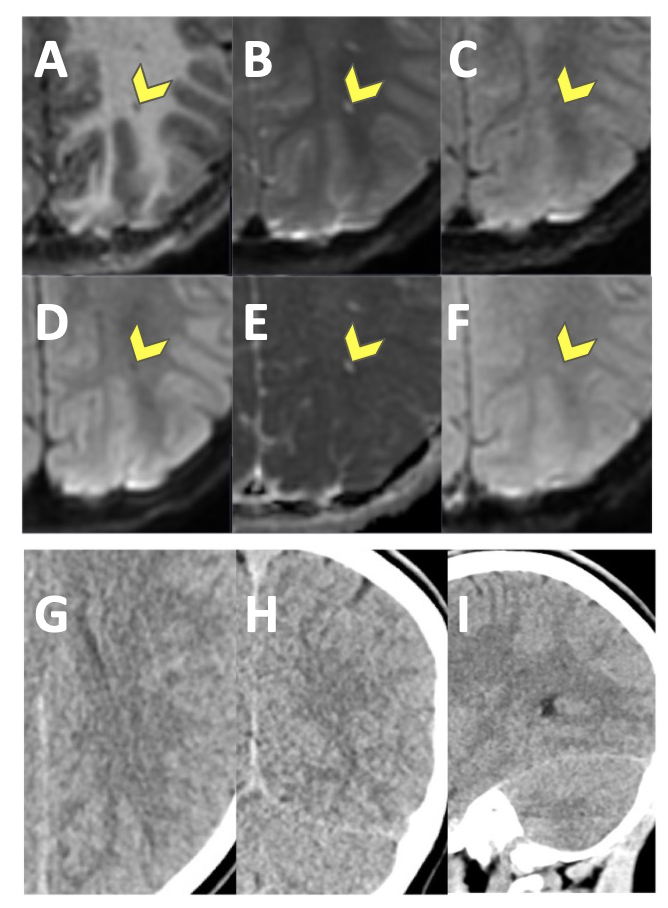


Supplementary Figure 4. ID10. Midline CSF-filled cavity (yellow head arrow), visible on both EPIMix and CT at the side-by-side consensus. T1-FLAIR (A), T2 (B), T2-FLAIR (C), DWI (D), ADC (E), T2* (F) EPIMIX images, and axial (G), coronal (H), sagittal (I) soft tissue window CT images.


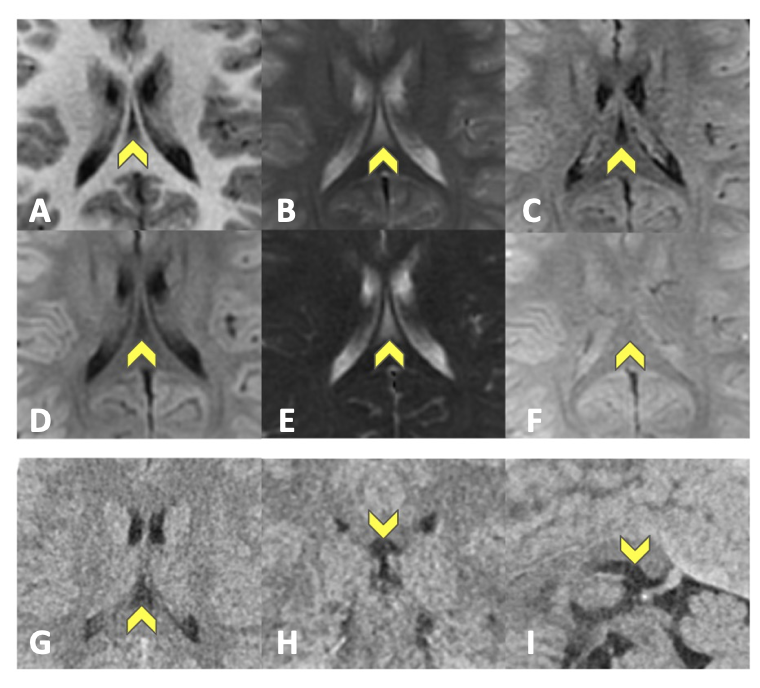


Supplementary Figure 5. One case of EPIMix evaluated as presenting “restricted” image quality due to distortion at the skull base region. The distortion in this specific case was due to a wrong frequency-encoding direction. T1-FLAIR (A), T2 (B), T2-FLAIR (C), DWI (D), ADC (E), T2* (F) EPIMIX images.


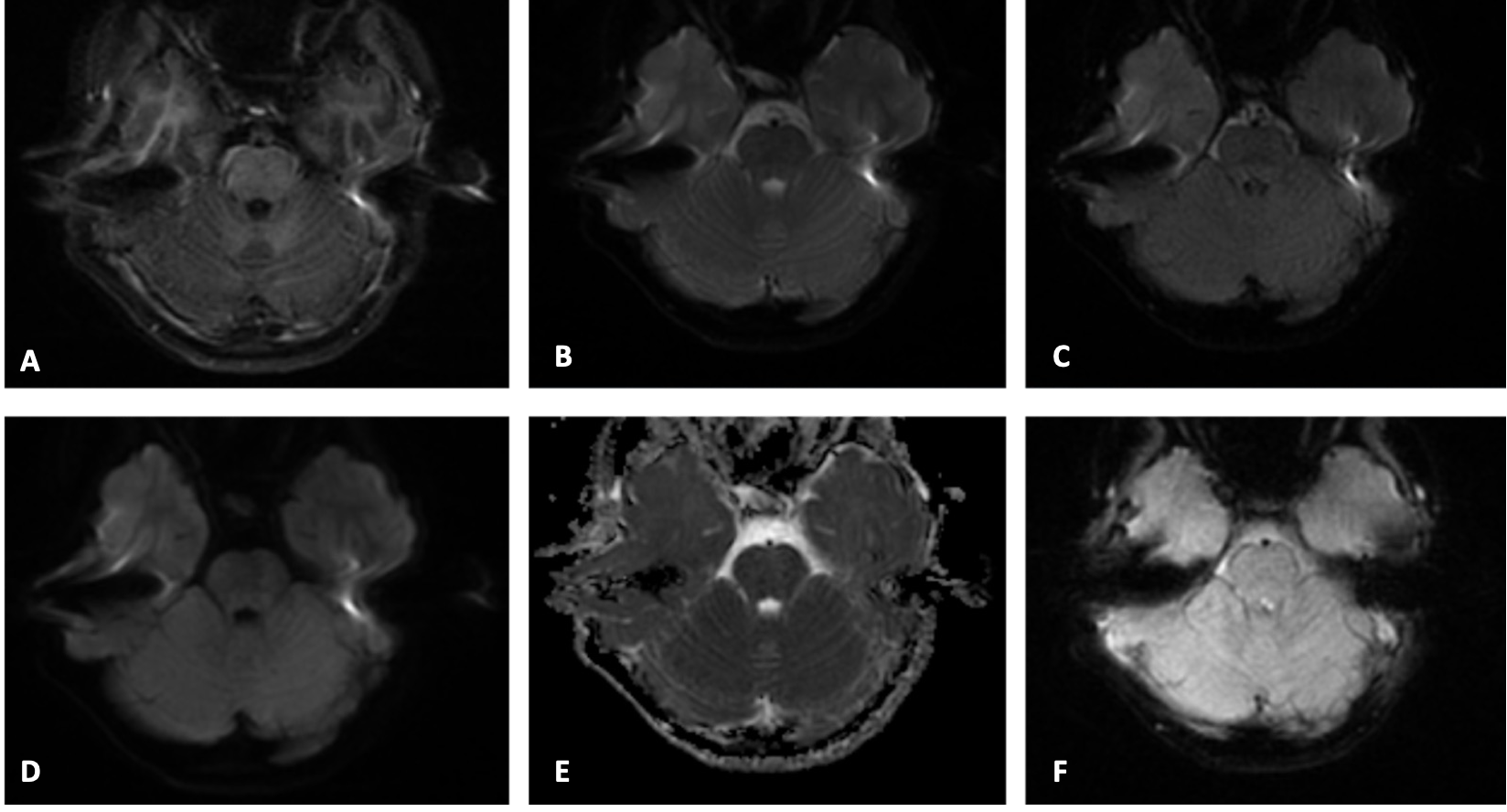


Supplementary Figure 6. ID 12. One case of EPIMix evaluated as presenting “restricted” image quality due to distortion in the posterior fossa. T1-FLAIR (A), T2 (B), T2-FLAIR (C), DWI (D), ADC (E), T2* (F) EPIMIX images.


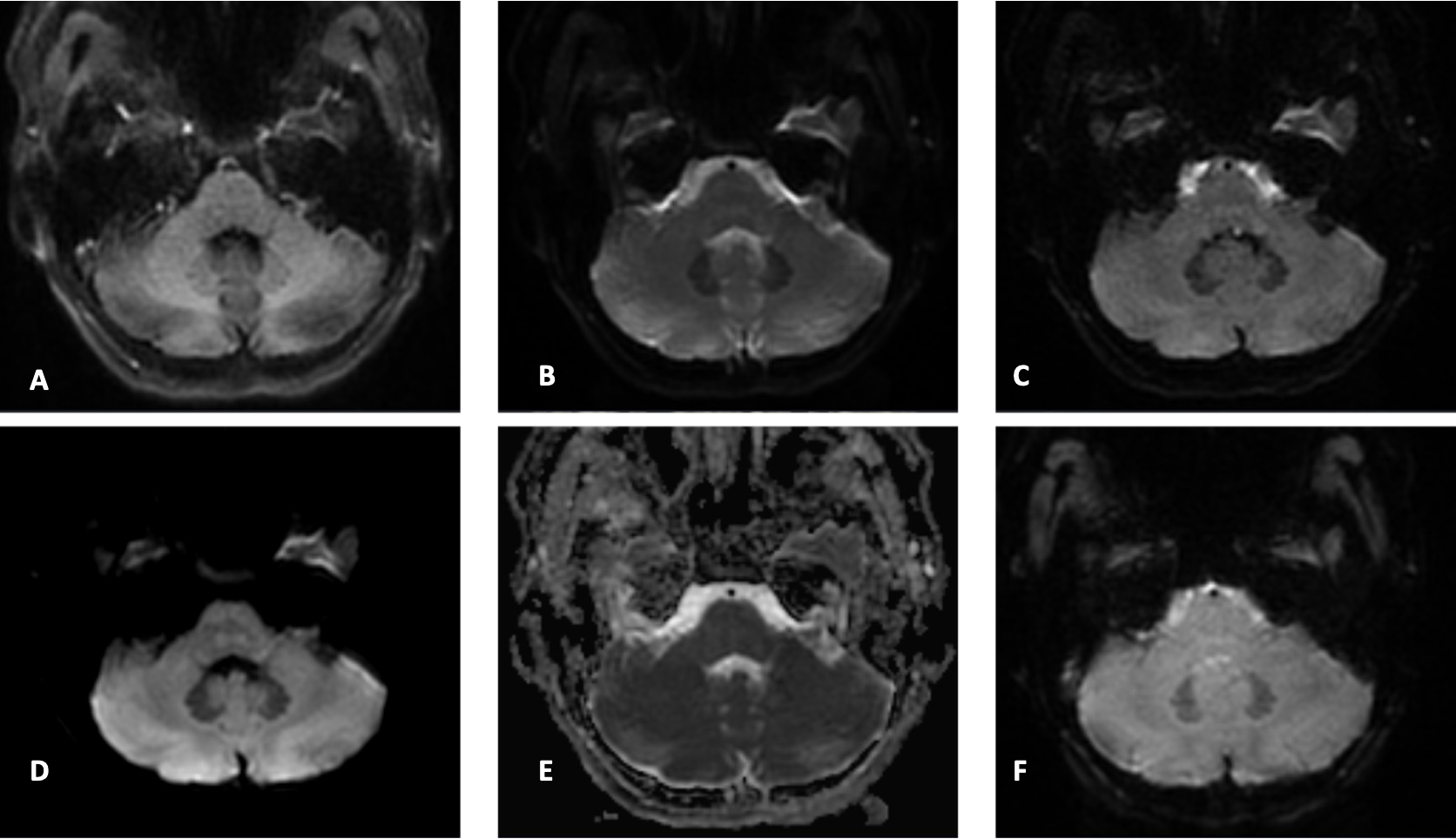


Supplementary Figure 7. Motion artifacts on EPIMix and CT scans on a per-reader (1–3) basis (CTR – CT reader, EPIMixR – EPIMixreader).


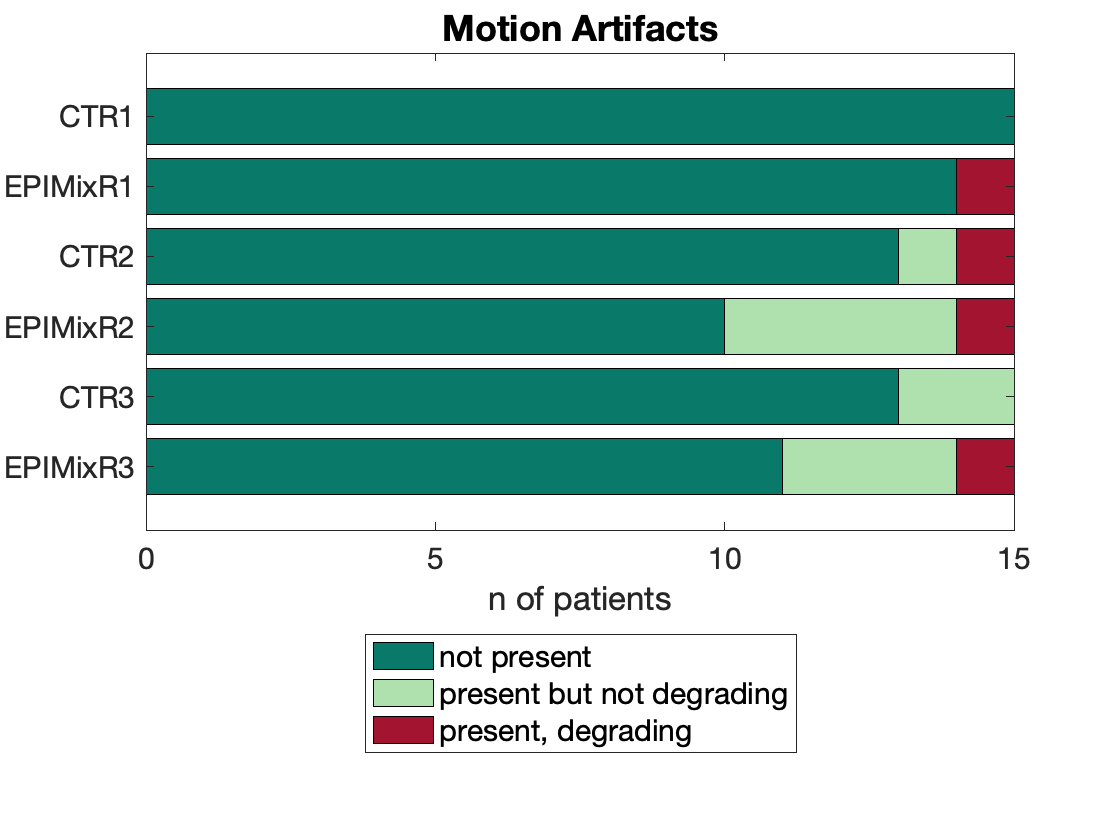


Supplementary Figure 8. Beam hardening artefacts on CT scans on a per-reader (1–3) basis (CTR – CT reader).


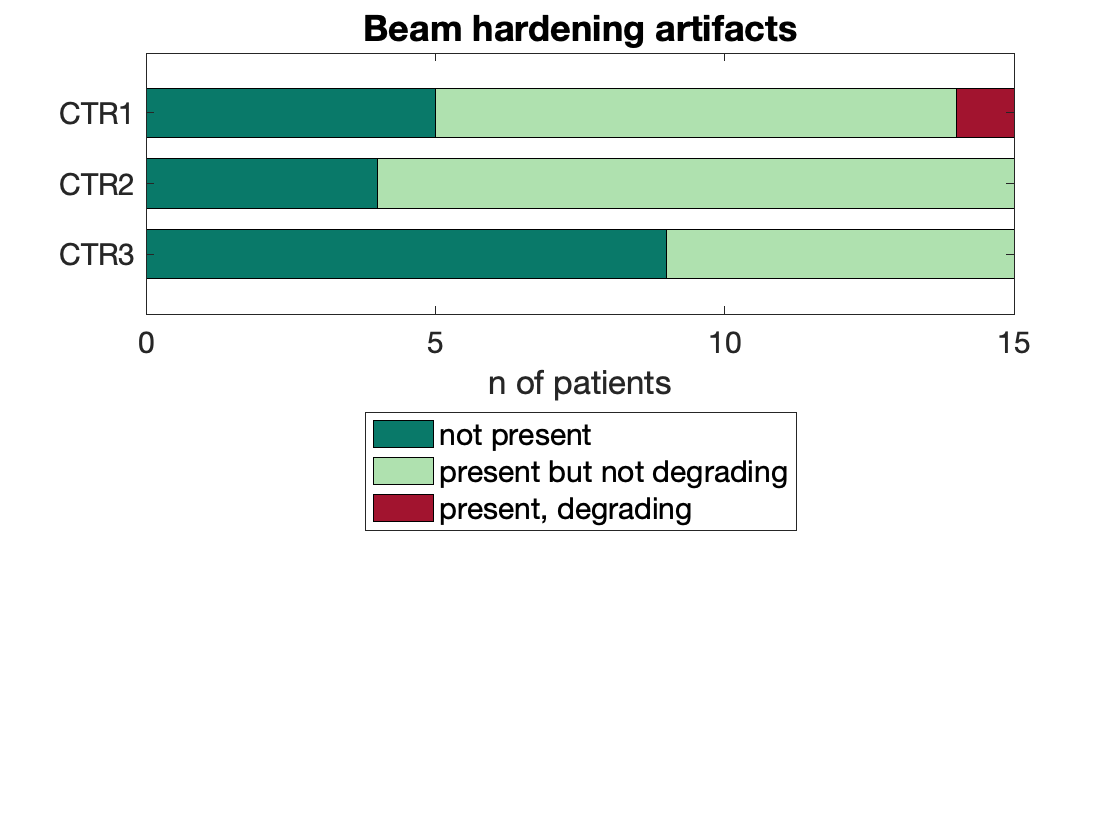


Supplementary Figure 9. Susceptibility distortion artefacts on EPIMix scans on a per-reader (1–3) basis (EPIMixR – EPIMixreader).


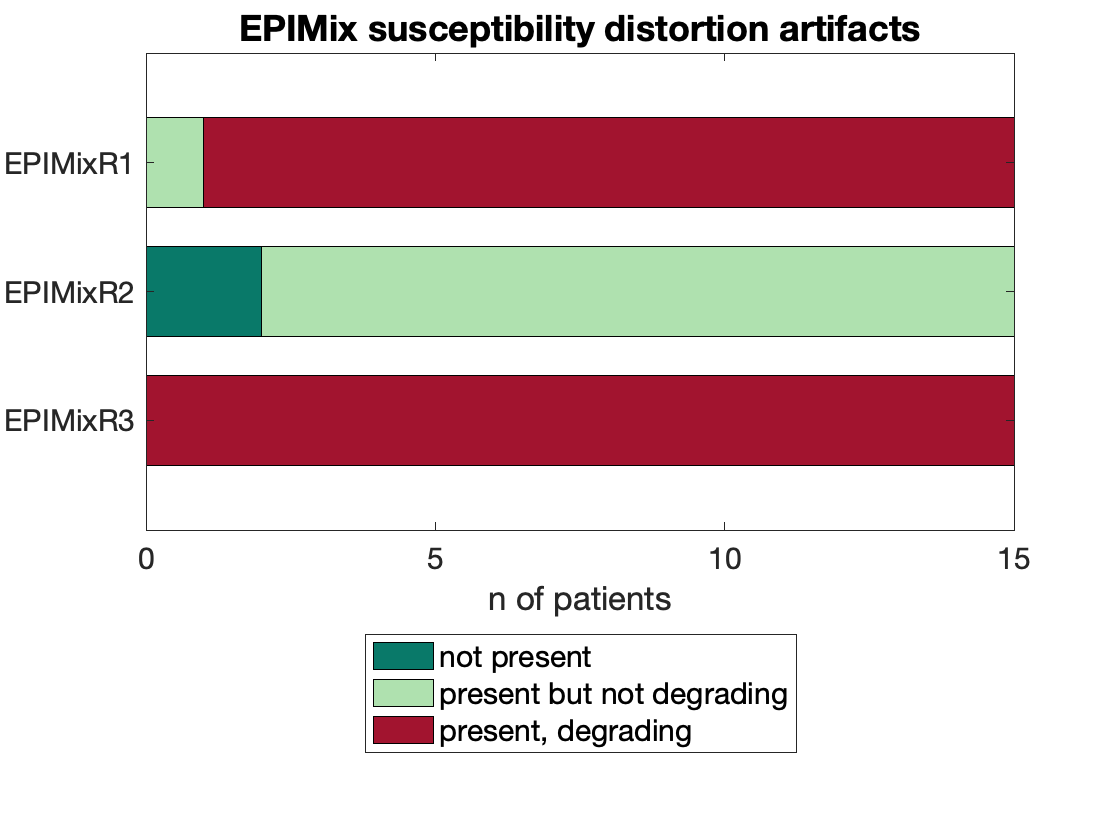

Supplement: Supplementary file 1 — Additional file 1. [file 12880_2024_1196_MOESM1_ESM.docx]
